# Evaluation of pharmacist’s practices regarding the antimicrobials dispensing: a simulated patient study

**DOI:** 10.1186/s12913-022-08853-y

**Published:** 2022-12-23

**Authors:** Elindayane Vieira de Souza, Lara Joana Santos Caxico Vieira, Sylmara Nayara Pereira dos Santos, Sabrina Cerqueira-Santos, Kérilin Stancine Santos Rocha, Rafaella de Oliveira Santos Silva, Divaldo Pereira de Lyra Jr

**Affiliations:** Laboratory of Teaching and Research in Social Pharmacy (LEPFS), Department of Pharmacy, Federal University of Sergipe, Avenue Marechal Rondon, Jd. Rosa Elze, São Cristóvão, Sergipe State 49100-000 Brazil

**Keywords:** Antimicrobials, Dispensing, Community pharmacies, Simulated patient

## Abstract

**Background:**

The indiscriminate use of antimicrobials is considered a major contributing factor to the increase in antimicrobial resistance. Community pharmacies are the main source of access to antimicrobials, and pharmacists are in a strategic position to promote rational use of these medicines. Therefore, it is important to know dispensing service quality.

**Objective:**

To evaluate the behavior of pharmacists in dispensing antimicrobials in community pharmacies in northeast Brazil.

**Methods:**

This cross-sectional pilot study was conducted from August to October 2021 in a private community pharmacy chain in Sergipe. Dispensing was evaluated using the simulated patient (SP) technique. Two SP asked the pharmacists for the antimicrobials (case clinic 1: upper respiratory infection; case clinic 2: urinary tract infection) and recorded the service through audio. Dispensing practices were independently analyzed by two researchers based on the tools available in the literature. Data were presented using descriptive statistics.

**Results:**

A total of 54 simulated patient visits were conducted. Based on the 12 steps recommended by the research team for good dispensing, pharmacists asked an average of 1 (±1.17) question for upper respiratory infections and 0.3 (±0.54) for urinary tract infections, as well as provided counseling (mean number of recommendations, 2.6 (±1.44) and 4.5 (±2.35), respectively). As for communication skills, pharmacists had a regular score (3.07 ± 0.34). Furthermore, there was no significant difference in the number of steps and counseling recommendations by pharmacists in dispensing clinical cases 1 and 2 (*p* = 0.0674).

**Conclusion:**

The quality of antimicrobial dispensing was evaluated as suboptimal, requiring improvements in practice and multifaceted strategies to promote continuing education of these professionals. In addition, awareness actions for the population must be implemented to promote the rational use of antimicrobials and reduce microbial resistance.

**Supplementary Information:**

The online version contains supplementary material available at 10.1186/s12913-022-08853-y.

## Introduction

Since their discovery, antimicrobials have contributed significantly to the reduction in morbidity and mortality associated with bacterial infections [1]. However, the global increase in their consumption is accompanied by antimicrobial resistance, which is considered one of the main threats to global health [2, 3]. According to the World Health Organization (WHO), approximately 35% of human infections are resistant to available antimicrobials. These data are even more alarming in low- and middle-income countries, with a resistance rate of up to 90% due to factors such as intense infectious disease burdens, regulatory enforcement, and limited essential resources for the diagnosis of diseases [4–6]. In addition, in these countries, inappropriate use of these medicines accounts for approximately 6 million deaths annually, with most cases being preventable [7].

Furthermore, approximately 93% of access to antimicrobials comes from community pharmacies, owing to their market penetration and geographic distribution [8]. Thus, community pharmacists are well-positioned to counsel the population [9]. Among the primary services provided by pharmacists, dispensing is highlighted as it serves the largest number of patients [10]. This clinical service guarantees the provision of medicines and other health products through analysis of the technical and legal aspects of the prescription (if applicable), assessment of individual health needs, and provision of interventions in the medication use process, including pharmaceutical counseling and practice documentation [11–13].

In Brazil, people have access to medicines through private community pharmacies and/or public pharmacies of the Unified Health System (SUS). In both cases, antimicrobials can only obtain through the prescription of a professional qualified to prescribe and dispensed by pharmacists or under their supervision [7, 8, 14]. Although there are regulations regarding the sale of antimicrobials, enforcement of and compliance with these measures is considered an old issue and difficult to operationalize in view of economic, cultural, information, education, and inspection factors [15, 16].

A recently published systematic review showed that drug dispensing has a positive impact on the clinical, humanistic, and economic outcomes of patients treated in community pharmacies [17]. Similarly, a narrative review indicated a significant improvement in the rational and appropriate use of antimicrobials after dispensing [18]. However, studies conducted in low- and middle-income countries have highlighted that antimicrobial dispensing practices are usually deficient [19]. In addition, most studies have investigated the sales of antimicrobials without prescription, with little emphasis on interventions provided during the service [20, 21]. Such studies may be improved by providing interventions that may be carried out during the dispensing process to reduce the indiscriminate use of antimicrobials and the rates of antimicrobial resistance.

Different methods have been used to assess antimicrobial dispensing practices in community pharmacies [18, 22, 23]. Among them, simulated patients (SPs) are commonly used as they can provide a methodical way of observing the behavior of pharmacists in actual practice [24]. This method helps in the formulation of educational strategies that address deficiencies in practice that will improve behavior and optimize services. Despite this, only a few studies in Brazil have used this method, and none have investigated the dispensing of antimicrobials [25–28]. Thus, this study aimed to evaluate the dispensing practices for antimicrobials in community pharmacies in northeast Brazil.

## Methods

### Study design

A cross-sectional pilot study was conducted from August to October 2021, using a simulated patient methodology. The study was reported in accordance with the ‘STROBE Statement—Checklist of items that should be included in reports of cross-sectional studies’ [29] and CRiSP Statement—Checklist for reporting research using simulated patient methodology [30].

### Study location and population

This study was conducted in a chain of private community pharmacies in Sergipe, Brazil. The study included pharmacists of both sexes aged over 21 years who worked in community pharmacies and who voluntarily agreed to participate. Pharmacists who could risk the anonymity of the SPs were excluded, that is, pharmacists who knew the simulated patients or those who suspected they were being evaluated (to avoid potential sources of bias). In this study, convenience sampling was performed; therefore, the sample size was not calculated, and pharmacists were selected according to their availability during the simulated visit.

### Simulated patients

#### Simulated patient method

The antimicrobial dispensing practices of the pharmacists were evaluated using SPs. In this method, SPs are specifically trained to simulate elaborate clinical cases identical to a genuine patient. This approach has been described in the literature as an effective method for obtaining objective and reliable data on pharmacists’ dispensing practices in a discrete manner [31–34].

The participating pharmacies invited their team of pharmacists to participate in the study to learn about the processes carried out by these professionals. Meetings were held between the research team and pharmacists, who were informed that their consultations would be audio-recorded and that the quality of drug dispensing would be evaluated. They were not informed when the simulated visits would occur, who the patients would be, or which clinical cases would be involved. The pilot test started 3 months after collecting data from the participants, and the actual simulated visits occurred 6 months after the pilot test.

The participants were also informed that the data obtained would be analyzed confidentially. Concurrently, the terms of consent were explained to the pharmacists who voluntarily agreed to participate in the study. Sociodemographic characteristics, such as age, sex, undergraduate institution (public or private), time since graduation, postgraduate studies, and time working in a community pharmacy, were collected. After 6 months, simulated visits were carried out for a pilot test and later official visits to participating pharmacies.

The visits followed a schedule agreed upon by the researchers, who determined the pharmacies to be visited and the itinerary to be followed. The dispensing was performed by the pharmacist present at the time of the simulated visit.

#### Training of SPs

The researchers selected two pharmacy students as SPs, and each was assigned to perform a clinical case to ensure consistency in the simulations (Additional file 1: Appendix A and B). The SPs were trained in a 2-hour workshop, which involved the following steps: presentation of the study objectives, scenario review, standardized scenario study, and instructions on behavior during the simulated visits. A pilot test was conducted with three pharmacists who did not participate in the study to check the functionality of the scenarios and assessment form and to standardize the behavior of SPs [31, 32]. A pre-test of the scenarios and assessment form was carried out by the SPs visiting a pharmacist who did not participate in the study.

#### Scenarios

Researchers have developed two scenarios through meetings based on SP studies and protocols/recommendations for the treatment of clinical conditions [34–36]. Two pharmacists with drug dispensing experience reviewed the clinical cases, and two physicians verified whether the cases represented common clinical scenarios. In the first clinical case, one SP used an antibiotic suspension for upper respiratory tract infection (URTI). In the second clinical case, another SP used antibiotics for urinary tract infection (UTI). These scenarios were developed to reflect the concerns of SPs with different needs. By signing a confidentiality agreement, the physicians who participated in the study voluntarily provided their prescriptions. The SPs were trained to act in a passive manner, only responding to questions from the dispensing pharmacist when asked about the situation. The SPs were instructed not to speak and to wait for counseling spontaneously; if the pharmacists did not provide counseling, the SP asked: “how to use this medication?” (Additional file 1: Appendix A). In addition, for clinical case 2, the simulated patient was instructed to ask the pharmacist: “I have taken antibiotics before and it gave me diarrhea; can this medication also cause diarrhea?” and “I take contraceptive pills; is there a problem?” (Additional file 1: Appendix B).

The SPs visited the pharmacies at various times between April and October 2021. They were instructed to seek care from a pharmacist, and, after the end of dispensing, they were instructed not to buy the medicine. Visits were audio-recorded on a mobile phone.

#### Evaluation of dispensing practices

Immediately after the visit, each SP evaluated the pharmacists’ communication skills using the instrument developed by Berger et al. [37]; the communication was translated and validated into Brazilian Portuguese by Mesquita et al. [26]. This instrument contains 23 structured items that assessed pharmaceutical counseling and communication skills. For this study, only 13 items assessing communication skills were used to evaluate the simulated visits. These items were answered using a 5-point Likert scale (1-poor, 2-poor, 3-fair, 4-good, 5-excellent). Both SPs received training on how to apply the instrument and performed three pilot tests for calibration. Furthermore, the SPs noted whether the pharmacist provided spontaneous counseling. If the pharmacist actively provided counseling without questions being asked, it was recorded as spontaneous counseling. If the pharmacist provided counseling only after the patient asked questions, it was recorded as nonspontaneous counseling.

To evaluate drug dispensing, the researchers reviewed the scenarios based on the clinical evidence for each clinical case and on the algorithm developed by Rocha et al. [38]. This algorithm presents the clinical reasoning involved in the drug dispensing process, as well as the main questions and counseling that can be provided during this practice. Therefore, the behavior expected of pharmacists in each case was reviewed, discussed, and agreed upon by the researchers according to this algorithm and relevant clinical protocols [34, 36]. From this discussion, 12 steps and 16 counseling recommendations were proposed by the research team for qualified dispensing (three counseling recommendations of the algorithm were not used because they did not apply to clinical cases). Two researchers independently analyzed the audio clips in a pre-formatted Excel spreadsheet. Discrepancies were resolved using a third rater.

### Statistical analysis

Data were tabulated and processed in spreadsheets created using Microsoft Excel and BioStat 5.3 softwares for Windows. The normality of continuous variables was verified using the Shapiro-Wilk test. The difference between clinical cases 1 and 2 of the SP with respect to the number of dispensing steps (questions and counseling) and communication skills was evaluated using the Wilcoxon signed-rank test.

To analyze the association between the factors (sex, age, training time, training institution, graduate studies, time working in the private community pharmacy chain) and the number of steps and counseling recommendations in dispensing, the Chi-square test was used. Cramer’s V was used to measure the strength of the association. This test was performed using a statistical software (http://vassarstats.net/newcs.html). The following values were used as a reference: Cramer’s V ≤ 0.2, weak association; 0.2 < Cramer’s V ≤ 0.6, moderate association; and Cramer’s V > 0.6, strong association. For all analyses, the confidence interval was 95%, and the differences were considered statistically significant at *p* ≤ 0.05.

### Ethical considerations

This study was approved by the Ethics Committee in Research Involving Human Beings of the Federal University of Sergipe – CEP/UFS under CAAE 15827719.4.0000.5546 (Register Number 3, 698, 806). All methods were performed in accordance with relevant guidelines and regulations. In addition, prior to data collection, participants were asked to sign the Free and Informed Consent Term e all research participants were informed about the objectives and voluntary nature of the study.

## Results

### General characteristics of the study participants

Twenty-seven community pharmacists participated in the study, and 54 visits were conducted; the pharmacists were identified with a badge and a specific uniform. The participants’ main characteristics are listed in Table 1. The average age of the pharmacists was 27.9 ± 4.4 years, the average training time was 3.5 ± 3.1 years, and only seven had (25.9%) completed postgraduate studies in the clinic.

**Table 1:** Characteristics of pharmacists working in private community pharmacies chain (*n* = 27)

Characteristics	Pharmacists (%)
Sex	
Female	21 (77.77%)
Male	6 (22.23%)
Age	
21-30 years	22 (81.49%)
31-40 years	4 (14.81%)
Greater than 41 years	1 (3.70%)
Training time	
Up to 1 year	4 (14.81%)
From 2 to 3 years	10 (37.03%)
From 4 to 5 years	11 (40.75%)
Greater than 5 years	2 (7.41%)
Training institution	
Public	11 (40.75%)
Private	16 (59.25%)
Postgraduate degree in clinical	
Yes	7 (25.93%)
No	20 (74.07%)
Time working at the company (years)	
Up to 1 year	8 (29.62%)
From 2 to 3 years	14 (51.85%)
From 4 to 5 years	5 (18.51%)

The dispensing steps performed by pharmacists during simulated visits are described in Table 2. With regard to the 12 steps recommended by the research team for qualified dispensing, pharmacists performed 1 step on average (minimum:0 | maximum:6) for clinical case 1 and 0.29 step (minimum:0 | maximum:2) for clinical case 2. In clinical case 1, three (11.11%) pharmacists sought to understand the SP’s other health needs, and one (3.70%) questioned whether the SP had undergone a diagnostic test for COVID-19. In clinical case 2, there were no additional questions regarding SP.

**Table 2:** Steps performed by pharmacists during simulated visits (*n* = 27)

Steps	Steps performed by pharmacists - n (%)	
	Clinical case 1 (URTI)	Clinical case 2 (UTI)
1. Checks the completeness and adequacy of the prescription according to current legislation	27 (100%)	27 (100%)
2. Checks whether dispensing is for the patient, caregiver or other person	18 (66.66%)	4 (14.81%)
3. Checks if it is the first time that the patient uses that medication	0	0
4. Checks for contraindications to the use of the drug: (eg allergy, pregnancy, health conditions, among others)	0	0
5. Checks if the patient uses other medications or has other health problems	1 (3.70%)	0
6. Checks if the patient knows the indication of the drug	1 (3.70%)	2 (7.40%)
7. Checks if the patient knows about the correct use of the drug	2 (7.40%)	0
8. Checks if the patient knows about the aspects related to the safety of the treatment (eg, have you ever felt anything different when using this medication?)	0	0
9. Checks if the patient is aware of aspects related to adherence to treatment (eg: Some people forget to take their medication, does this happen to you? What are your concerns about the use of this medication?	0	1 (3.70%)
10. Confirms the patient's understanding of the guidelines provided in dispensing	1 (3.70%)	0
11. Documents the interventions performed in the dispensation	0	0
12. Refers the patient to other healthcare professionals and/or pharmaceutical services when necessary	^*^	^*^
Total number of times the steps were performed by pharmacists	**50**	**34**

^*^step does not apply to clinical cases, according to the judgment of the research team

Pharmaceutical counseling provided during the simulated visits to clinical cases 1 and 2 is shown in Table 3. The average number of counseling recommendations per simulated visit was 2.6 ± 1.4 for clinical case 1 and 4.5 ± 2.3 for clinical case 2. For clinical case 1, pharmacists provided spontaneous counseling in only six (31.0%) simulated visits, and only three (11.1%) pharmacists provided counseling on how to prepare the suspension. For clinical case 2, the pharmacists provided spontaneous counseling in only two (7.4%) simulated visits.

**Table 3:** Counseling provided by pharmacists during simulated visits (*n* = 27)

Counseling provided	Counseling provided by pharmacists - n (%)	
	Clinical case 1	Clinical case 2
1. Inform the name of the drug	18 (66.66%)	13 (48.14%)
2.Provides counseling on the clinical condition (disease/sign/symptom) for which the drug was prescribed	1 (3.70%)	2 (7.40%)
3. Counseling about drug indication	2 (7.40%)	7 (25.92%)
4. Counseling about therapeutic goals and/or goals	*	*
5. Counseling about how to use the pharmaceutical form of the drug	3 (11.11%)	2 (7.40%)
6. Informs the route of administration of the drug	2 (7.40%)	0
7. Counseling about posology (dose, frequency and duration of treatment)	26 (96.29%)	29 (100%)
8. Counseling about the time of drug administration	7 (25.92%)	10 (37.03%)
9. Counseling about time for drug to take effect	0	0
10. Counseling about interactions (drug/drug; drug/food; drug/alcohol)	0	18 (66.66%)
11. Counseling about how to monitor health problem	*	*
12. Counseling about non-pharmacological treatment	0	6 (22.22%)
13. Counseling about medicine storage	6 (22.22%)	0
14. Counseling about disposal of the drug	1 (3.70%)	6 (22.22%)
15. Counseling about precautions regarding drug use (e.g. for drugs that cause drowsiness, be extra careful when driving or operating machinery)	2 (7.40%)	15 (55.55%)
16. Counseling about adverse drug reactions	0	13 (51.85%)
17. Counseling about consequences of long-term drug use, where applicable	*	*
18. Counseling about action in case of missed dose	0	0
19. Counseling about importance of correct use and adherence to treatment	3 (11.11%)	4 (14.81%)
Total number of times the steps were performed by pharmacists	**71**	**125**

^*^counseling does not apply to clinical cases, in the judgment of the research team

There was no significant difference between the number of steps and counseling recommendations followed by pharmacists in dispensing for clinical cases 1 and 2 (Z = 1.829; *p* = 0.067). In addition, the number of steps and counseling recommendations followed during dispensing of medications for clinical cases 1 and 2 was not significantly associated with sex (χ^2^ = 0.652; *p* = 0.722; Cramer’s V = 0.155), age (χ^2^ = 5.444; *p* = 0.244; Cramer’s V = 0.317), training time (χ^2^ = 8.593; *p* = 0.19; Cramer’s V = 0.398), training institution (χ^2^ = 2.716; *p* = 0.257; Cramer’s V = 0.317), graduate degree (χ^2^ = 2.419; *p* = 0.298; Cramer’s V = 0.299), or time spent working in the private community pharmacy chain (χ^2^ = 5.251; *p* = 0.262; Cramer’s V = 0.311). In four (14.8%) visits, pharmacists advised on the importance of correct use and adherence to antimicrobial treatment due to antimicrobial resistance.

The results for the communication skills are presented in Table 4. The pharmacists only dedicated enough time on 15 (55.5%) visits (approximately 5 min well-spent) to patient care. During six (22.2%) visits, pharmacists provided written counseling. No pharmacist provided counseling on what to do in case of doubt.

**Table 4:** Assessment of pharmacists’ communication skills during the antimicrobials dispensing

Evaluation criteria	Clinical Case 1 URTI (*n* = 27)				Clinical Case 2 UTI (*n* = 27)				Total (*n* = 54)		p
	Min	Max	Average	SD	Min	Max	Average	SD	Average	SD	
1. Reception (reception)	1	5	3.00	0.83	1	5	2.92	0.95	2.83	0.74	0.7174
2. Visual contact	2	5	3.29	0.77	2	5	3.44	0.64	3.32	0.68	0.3078
3. Interest (attention)	2	5	3.48	0.80	2	5	3.33	0.68	3.38	0.73	0.4229
4. Involvement	1	5	3.37	0.92	2	5	3.26	0.76	3.28	0.84	0.5695
5. Farewell	1	5	3.07	0.87	2	4	3.14	0.81	3.10	0.87	0.7938
6. Availability	1	5	2.59	0.97	1	4	2.07	0.61	2.32	0.85	0.0217*
7. General impression of non-verbal communication	2	5	3.03	0.75	2	5	3.07	0.67	3.02	0.69	0.8139
8. Logical sequence of orientation	1	5	3.07	0.82	3	5	3.44	0.64	3.26	0.78	0.0383*
9. Emphasis on important aspects	1	5	3.07	0.99	2	5	3.18	1.03	3.16	0.98	0.6417
10. Use of terms appropriate to the patient's level of understanding	2	5	3.44	0.69	3	5	3.33	0.55	3.38	0.63	0.4802
11. Length of prayers (very long prayers can compromise understanding)	2	5	3.25	0.76	2	4	3.00	0.48	3.16	0.62	0.0745
12. Use of open-ended questions, which make room for the patient to speak	1	5	2.51	0.93	1	4	2.26	0.98	2.44	0.95	0.2598
13. General impression of the service	1	5	3.07	0.78	1	5	2.96	0.94	3.02	0.82	0.5049

^*^Value de *p* ≤ 0.05

## Discussion

This study investigated the practices of pharmacists regarding the dispensing of antimicrobials in community pharmacies in Sergipe, Brazil, showing deficiencies in the dispensing process, such as asking sufficient questions to assess the SPs’ needs and, consequently, providing appropriate recommendations. Similarly, other studies have reported that most pharmacists dispensed antimicrobials without asking about the previous use of the requested medication, allergy history, or associated symptoms [39, 40]. The literature indicates that several factors can contribute to this scenario, such as the characteristics of the SP’s case, pharmacists’ lack of time or knowledge, and intrinsic patient factors [22, 34, 37, 41]. Obtaining information during dispensing is essential for decision making and adequate counseling [38]. Therefore, it is important that strategies are developed so that pharmacists can adopt well-defined work processes.

Most pharmacists provided a limited amount of counseling for both clinical cases, which could compromise the patients’ proper use of antimicrobials. Pharmaceutical counseling is an essential component of dispensing, even though there are factors that may influence its quantity and quality [38]. Pharmacist training may be one such factor, as suggested by a study in Republika Srpska, Bosnia, and Herzegovina, in which training initiatives reduced the rate of antimicrobial dispensing without a prescription in community pharmacies from 58 to 18.5% [42, 43]. This finding is similar to that of other studies showing that pharmacist training initiatives reduce inappropriate antimicrobial dispensing and should be continued. But there are also contrary study results in the international literature, showing that interventions are not always successful [44–46]. Thus, the adoption of multifaceted strategies to improve dispensing practices is key to promoting rational use of antimicrobials.

In general, SPs in clinical case 2 (UTI) received more instructions than SPs in clinical case 1 (URTI). This can be explained by the scenario itself, in which the patient in clinical case 2 exhibited a more active behavior. The study by Alaqeel and Abanmy [39] supports this finding, wherein they observed a referral rate of 3%, which improved to 43% when SPs requested additional information. This attitude is consistent with the literature, which reports an association between a patient’s active behavior and the provision of more information [47, 48]. Additionally, patients should be encouraged to take an active role in developing their autonomy and self-care and, consequently, their co-responsibility for their health [49, 50]. Thus, pharmacist-patient communication should be stimulated.

In this study, a limited spontaneous counseling rate was observed in both the clinical cases. In contrast, a study carried out in Slovakia found higher rates of spontaneous counseling (55.6%) of visits [51]. The literature shows that counseling rates are influenced by factors such as the complexity of the clinical case, time limitations, intense flow of people, knowledge of the pharmacist, and attitude of patients [48, 52]. A study conducted in Asia highlighted that inadequate clinical experience and insufficient knowledge of antimicrobials are the main factors for deficient antimicrobial dispensing [52]. However, without counseling, the interaction between the pharmacist and patient is reduced to a commercial transaction [51]. Thus, a proactive attitude should be encouraged among pharmacists to improve the quality of their practice.

The pharmacists’ communication skills were generally evaluated as regular. These data are superior to those of a previous study conducted in Sergipe, which showed that pharmacists had low scores for these skills [26]. Communication skills contribute to the establishment of a pharmacist-patient therapeutic relationship, which is necessary for the provision of quality health care [53]. Furthermore, such skills, added to the quality of pharmaceutical counseling provided to patients, have the potential to increase user loyalty to pharmacies [54]. Thus, improving competence can be a strategy for improving the quality of care.

Therefore, several factors can be identified as causing deficiencies in pharmacist dispensing practices. For instance, their academic training may be deficient in defined work processes, thus hindering efficient dispensing. Studies have indicated that the absence of systematized and reproducible work processes in dispensing results in inconsistent pharmaceutical interventions in clinical practice [55, 56]. Therefore, it is necessary to develop training strategies to ensure that pharmacists follow the best standards of patient care.

Moreover, a high number of patients to use community pharmacies as a first resource for obtaining medicines and correlates, may compromise the quality of dispensing of medicines, because pharmacists may feel pressured to provide a fast service that can serve a greater number of people without the provision of quality pharmaceutical counseling. In addition, studies report that patients often distort the role of the pharmacist and do not view community pharmacies as health units, with their view of the pharmacy as a provider of rapid care [57–59].

## Limitations

Although repeated training was carried out to ensure consistency between the SPs’ performance and to increase the internal validity of the collected data, it is still possible that some interpersonal differences have occurred, especially regarding the subjective items of communication skills. In addition, a real patient has the tendency to communicate freely about his/her pathology; therefore, the results measured by this method may differ in real situations. Furthermore, the application of the consent form and convenience sampling adopted in this study may have caused a selection bias. Thus, the small number of participants in this study may have affected the results, suggesting caution when generalizing these results.

## Conclusion

This study shows that antimicrobial dispensing practices in community pharmacies are insufficient. Most pharmacists did not ask questions to assess the health needs of the SPs. The counseling rate was low, and pharmacists provided more counseling upon request for information. In general, the pharmacists’ communication skills were considered fair. Policymakers, pharmacy managers, and pharmacists must create strategies to improve antimicrobial dispensing practices. Therefore, patient education and continuing professional education be implemented to promote the rational use of antimicrobials.

## Supplementary Information

**Additional file 1: Appendix A.** Case 01 of Simulated /Patient. **Appendix B.** Case 02 of Simulated Patient.

## Data Availability

The datasets used and/or analyzed during the current study are available from the corresponding author upon reasonable request.

## Abbreviations

COVID-19: Coronavirus Disease 2019
SP: Simulated Patient
URTI: Upper Respiratory Tract Infection
UTI: Urinary Tract Infection
WHO: World Health Organization
