# Supplementary material for: Evaluation of pharmacist’s practices regarding the antimicrobials dispensing: a simulated patient study
Source: BMC Health Serv Res. 2022 Dec 23;22:1576. doi: 10.1186/s12913-022-08853-y (PMC9788868; doi:10.1186/s12913-022-08853-y)
Supplement: Supplementary file 1 — Additional file 1: Appendix A. Case 01 of Simulated /Patient. Appendix B. Case 02 of Simulated Patient. [file 12913_2022_8853_MOESM1_ESM.docx]

**APPENDIX A - Case 01 of Simulated /Patient**

**Upper Respiratory Tract Infection (URTI)**

| **Scenario 01: Upper Respiratory Tract Infection** |
| --- |
| Woman, 22 years old, enters the pharmacy, asks the pharmacist and delivers the prescription with the following medications:  Amoxicilline (suspension) (250 mg/5mL), take 10mL 8/8h daily (1-1-1) for 10 days.  Ibuprofen drops 100mg/mL, take 40 drops if pain |

| **SCRIPT FOR THE SIMULATED PATIENT - CASE 1** | |
| --- | --- |
| You went to the doctor complaining of fever two days ago, inflammation of the throat, swallowing pain, headache, and fever (38ºC) (tonsillin exudation, inflamed tonsillium). Absence of other symptoms such as cough, runny nose, sneezing, loss of taste. Other health problems, nor does he use other medicines. No history of recurrent infections.  The drug for respiratory infection (pharyngitis) (Ibuprofen drops 100mg/mL, 40 drops, if pain + Amoxicilline (suspension) (250 mg/5 mL), take 10mL 3x daily (1-1-1) for 10 days was prescribed. It was passed suspension because you have difficulty swallowing large pills. You're a passive, pleasant, shy patient.  When you enter the pharmacy, you ask the pharmacist: " How to use this medication?" | |
| **Pharmacist question** | **Answer** |
| Do you have registered Individual Taxpayer Identification Number? | Yes |
| Who's the medicine for? | For myself |
| Is there a history of allergy? | No |
| Is this the first time you will use this medicine? | "I think so. I've had antibiotics before, but I can't remember the name." |
| How did this infection come about? | "This climate change got me" |
| Did you take the COVID-19 test? | I did, but I'm waiting for the results. But the doctor thinks it's a throat infection. |
| What symptoms are you experiencing? | "I have a sore throat, it hurts to swallow my saliva, I have a fever, I have a headache”  *If asked about fever, the temperature was on average 38ºC |
| Are there other symptoms (cough, runny nose, sneezing, loss of taste, etc.) or do you have another disease? | No |
| Do you use other medications? | No, I don't like taking medicine very much |
| Have you used other medications prior to this treatment? | No. I just garbled with warm water and salt |
| Do you know the indication of the medicine? | Yes, it's for throat infection |
| Do you know how to use the medicine? | The patient will look at the prescription and say "… No. The doctor did, but I don't remember. It was very fast." |
| Do you know the aspects related to treatment safety? | No |
| If asked about lifestyle: | You don't usually drink a lot of water (less than 2L per day)  Sedentary  Unbalanced feeding  Stress (currently in college)  Frequents air-conditioned environment  You don't drink, you don't smoke |
| Note. If the pharmacist asks to look at the throat, patient should refuse. For example, "Do you really need it? I just came from the doctor, and he's already looked." | |
| **If the pharmacist does not question or guide anything the patient should ask how he will use the medicine.** | |
| **If the pharmacist dispenses:**  Patient goes to the cashier, but does not make the purchase, because realizes that is without the credit card. Patient is surprised. “Can you cancel the purchase? I forgot my wallet at home."  Patient apologizes for the inconvenience and thanks. | |

**APPENDIX B- Case 02 of Simulated Patient**

**Urinary Tract Infection (UTI)**

| **Scenario 02: Urinary Tract Infection (cystitis)** |
| --- |
| Woman, young, 23 years old, enters the pharmacy, requests the pharmacist, and delivers the prescription Macrodantina (nitrofurantoin) 100mg, 6h/6h for 5 days + Pyridium (Phenazopyriride hydrochloride) 200mg, 8h/8h, for 2 days. |

| **SCRIPT FOR THE SIMULATED PATIENT - CASE 2** | |
| --- | --- |
| You go into the pharmacy and ask the pharmacist. Delivery to him/her the prescription of Macrodantina (nitrofurantoin) 100mg, 6h/6h for 5 days + Pyridium (Phenazopyriride hydrochloride) 200mg, 8h/8h, for 2 days. You have a urinary tract infection, feeling pain and ardor when urinating and wanting to go to the bathroom all the time, since yesterday (dysuria, increased urinary frequency, urinary urgency, suprapubic pain). There is no presence of other symptoms such as bad smell and discharge. It has no other health conditions. Medication in use: Repopil contraceptive (cyproterone acetate + etinilestradiol).  You went to the doctor; however, the service was very fast, he just asked a few questions and passed these medications. You're a nice, nice patient who asks some questions.  When entering the pharmacy, you ask the pharmacist: "I want these medicines, please" | |
| **Pharmacist question** | **Answer** |
| Do you have registered Individual Taxpayer Identification Number? | Yes (Provides the number) |
| Who's the medicine for? | For myself. |
| Is there a history of allergy? | No |
| There is contraindication (Are you pregnant or breastfeeding?) | No, thank God |
| Is this the first time you will use this medicine? | "I think so. I've had urinary tract infection for a long time (+ 1 year), but I can't remember the treatment" |
| What symptoms are you experiencing? | "I'm in pain and it's been in pain and I'm dying to pee, and I want to go to the bathroom all the time since yesterday" |
| Are there other symptoms (bad smell, discharge) or other diseases? | No. Just those symptoms anyway. |
| Do you use other medications? | yes, birth control.  *If asked about the contraceptive, the patient takes Repopil.  If the pharmacist does not ask, the patient will ask "I take contraceptives, do you have a problem with this medication?" |
| Do you know the indication of the medicine? | Yes, it's for urinary tract infection. |
| Do you know how to use the medicine? | The patient will look at the prescription and say "… No. The doctor did, but I don't remember. It was very fast." |
| Do you know the aspects related to treatment safety? | Don't know  If the pharmacist does not ask, the patient will ask:  "Once I took an antibiotic and gave diarrhea. Can this also cause?" |
| If asked about lifestyle: | Ingests little water (less than 2L per day)  Wears a lot of jeans (pants) (student)  It usually traps the pee  Does physical activity  Rule-of-power  Drink alcohol socially (2x a week), do not smoke  Active sex life (one partner) |
| **If the pharmacist does not question anything and/or guide, you should ask how you will use the drug and question whether this drug can cause diarrhea, because once you used an antibiotic and gave diarrhea, but you do not remember the name and if you have problem taking this medication with the contraceptive.** | |
| **If the pharmacist dispenses:**  Patient goes to the cashier, but does not make the purchase, because realizes that is without the credit card. Patient is surprised. "Can you cancel the purchase? I forgot my wallet at home."  Patient apologizes for the inconvenience and thanks. | |
